# Phenotype of Transgenic Mice Overexpressed with Inducible Nitric Oxide Synthase in the Retina

**DOI:** 10.1371/journal.pone.0043089

**Published:** 2012-08-08

**Authors:** Guey Shuang Wu, Meisheng Jiang, Yi-Hsin Liu, Yoshiko Nagaoka, Narsing A. Rao

**Affiliations:** 1 Department of Ophthalmology, Doheny Eye Institute, Keck School of Medicine, University of Southern California, Los Angeles, California; 2 Department of Molecular and Medical Pharmacology, David Geffen School of Medicine, University of California Los Angeles, Los Angeles, California; University of Florida, United States of America

## Abstract

**Background:**

Unlike its constitutive isoforms, including neuronal and endothelial nitric oxide synthase, inducible nitric oxide synthase (iNOS) along with a series of cytokines are generated in inflammatory pathologic conditions in retinal photoreceptors. In this study, we constructed transgenic mice overexpressing iNOS in the retina to evaluate the effect of sustained, intense iNOS generation in the photoreceptor damage.

**Methods:**

For construction of opsin/iNOS transgene in the CMVSport 6 expression vector, the 4.4 kb Acc65I/Xhol mouse rod opsin promoter was ligated upstream to a 4.1 kb fragment encoding the complete mouse cDNA of iNOS. From the four founders identified, two heterozygote lines and one homozygote line were established. The presence of iNOS in the retina was confirmed and the pathologic role of iNOS was assessed by detecting nitrotyrosine products and apoptosis. Commercial TUNEL kit was used to detect DNA strand breaks, a later step in a sequence of morphologic changes of apoptosis process.

**Results:**

The insertion and translation of iNOS gene were demonstrated by an intense single 130 kDa band in Western blot and specific immunolocalization at the photoreceptors of the retina. Cellular toxicity in the retinas of transgenic animals was detected by a post-translational modification product, tyrosine-nitrated protein, the most significant one of which was nitrated cytochrome c. Following the accumulation of nitrated mitochondrial proteins and cytochrome c release, marked apoptosis was detected in the photoreceptor cell nuclei of the retina.

**Conclusions:**

We have generated a pathologic phenotype with sustained iNOS overexpression and, therefore, high output of nitric oxide. Under basal conditions, such overexpression of iNOS causes marked mitochondrial cytochrome c nitration and release and subsequent photoreceptor apoptosis in the retina. Therefore, the modulation of pathways leading to iNOS generation or its effective neutralization can be of significant therapeutic benefit in the oxidative stress-mediated retinal degeneration, a leading cause of blindness.

## Introduction

There are three known isoforms of nitric oxide synthase and all three isoforms generate nitric oxide (NO) by the catalytic conversion of arginine to citrulline [1]. Endothelial nitric oxide synthase (eNOS) and neuronal nitric oxide synthase (nNOS) are restricted to the defined subcellular domains and require calcium and calmodulin for their activation. These two isoforms are constitutively present to generate a small amount of NO for physiological functions [1], [2]. On the contrary, iNOS is an inflammation responsive enzyme that is calcium/calmodulin-independent [1]–[3]. An excessive amount of NO generation [3] by iNOS in the pathologic conditions is elicited by immune system activators, such as endotoxins and the cytokines, including interleukin-I (IL-1), interleukin-6 (IL-6), and tumor necrosis factor-α (TNF-α) [4]–[6]. Therefore, the *in vivo* function of iNOS is invariably tied to the inflammatory systems, in which iNOS is known to accentuate T cell proliferation and to increase the production of pro-inflammatory cytokines [7]. Nitric oxide, once produced rapidly scavenges the superoxide to form the potent biological oxidant peroxynitrite, which is known to cause irreversible tissue damage [7]. With this known detrimental potential, iNOS-toxicity has been found in several ocular inflammatory diseases [4] as well as in neuropathological diseases with marked inflammatory components, such as mutiple sclerosis, Parkinson's disease, and the early stages of Alzheimer's disease [8].

Consistent with the current trend of using the gene knockouts (KOs) to evaluate the function of target genes, iNOS KO has frequently been used in recent studies [9]. In this laboratory, a series of experiments was performed to investigate the role of iNOS in the early stage of experimental autoimmune uveoretinitis (EAU). Deletion of iNOS gene prevented oxidative stress and simultaneously abrogated the peroxynitrite-mediated tyrosine nitration in the retinal photoreceptors in EAU. While these results suggest a causative role of iNOS in retinal pathology, the specific contribution of upregulated iNOS expression isolated from that of inflammatory cytokines was not evaluated [10].

Further, using cardiovascular systems, the respective functions of all three isoforms of NOS have been investigated in pharmacological studies with specific NOS inhibitors and also in studies with mice that lack iNOS isoforms. These studies concluded that there were always some elements of uncertainty, such as in pharmacological studies, the specificity of the NOS inhibitors continued to be an issue of debate, and while in each type of the NOS isoform-deficient mice, compensatory effects by other NOS isoform were frequently encountered [11].

Intraocular injections of commonly used NO donors have been reported in rats; similar reports in mice, however, are scarce. An intravitreal injection of N-ethyl-2-(1-ethyl-2-hydroxy-2-nitrosohydrazino) ethanamine (NOC12) causes cell loss in GCL and thinning in IPL and INL, but no effect on ONL. NOC-12 is known to release a larger amount of NO spontaneously. Other studies using S-nitroso-N-acetylpenicillamine (SNAP) failed to demonstrate any retinal toxicity [12]. Further, some typical NO donors, such as sodium nitroprusside (SNP) are known to have co-factor requirement for NO release and also some biological activity in themselves [13]. Therefore, with these potential problems reported, it would be difficult to deliver NO to a defined area in the retina and retain a sufficient local concentration of NO in that area for a long period of time.

As *in vivo* toxicity of iNOS is shown to be invariably tied to the immune system activaters [4]–[6], and in the evaluation of toxicity in each type of the NOS-deficient mice, the compensatory effect by other isoform has always known to occur [11]. In this study, we used iNOS overexpressed transgenic mice to single out the effect of iNOS. In this iNOS knockin system, the contribution of cytokines is totally excluded from the system. Generation of such an *in vivo* system to study effect of iNOS has not been attempted in the past. Using this novel approach, we investigated whether constitutively overexpressed iNOS alone would cause tissue damage. Such a damaging effect will be derived strictly from the sustained signaling of endogenous iNOS.

## Materials and Methods

### Generation of transgenic mice overexpressing iNOS, specifically in the retinal photoreceptors

This study was carried out in strict accordance with the recommendations in the Guide for the Care and Use of Laboratory Animals of the Association of Research in Vision and Ophthalmology. The protocol was approved by the Committee of the Ethics of Animal Experiments of the University of Southern California (Protocol Number: 11218). All surgery was performed under ketamine and xylazine anesthesia, and all efforts were made to minimize suffering of animals.

**Figure 1:**
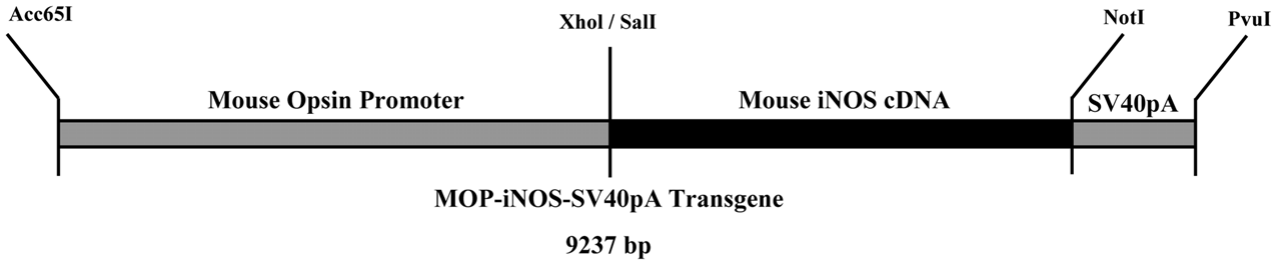
Map of opsin promoter-driven iNOS transgene used for generation transgenic mice with retinal iNOS-overexpression. The 4.4 kb Acc65I/Xhol mouse opsin promoter fragment was isolated and cloned into the Acc65I/Sall sites of pCMVSport6-iNOS. The polyadenylation signal was provided by the existing SV40 polyadenylation sequence which was immediately downstream of the iNOS cDNA. To release the transgene from the vector for microinjection, a double restriction enzymatic digestion employing Acc65I and Pvul was performed.

The opsin/iNOS transgene was constructed by ligating the 4.4 kb Acc65I/XhoI mouse rod opsin promoter upstream of the 4.1 kb fragment encoding the complete mouse cDNA of iNOS in pCMVSport6 expression vector (MGC mouse verified FL clone, mouse NOS2; Open Biosystems, Huntsville, AL). The mouse opsin promoter in pBluescript SKII was a kind gift from Dr. J. Chen (Cell and Neurobiology, University of Southern California) [14]. The transcriptional termination signal was supplied by the simian virus 40 polyadenylation site, producing a 9.2 kb fusion gene construct (Fig. 1). Prior to pronuclei injection, this 9.2 kb Acc65I/PvuI fragment was purified free of vector sequences. The parental CMV-driven iNOS expression plasmid was transfected into fetal retinal pigment epithelial cells for testing the protein expression efficiency before pronuclei injection. Gel purified opsin/iNOS transgene was injected into the fertile, one cell mouse embryos from either B6D2F1, or C57BL/6 (Charles River Laboratories, Wilmington, MA). Pronuclei injection was carried out by the Transgenic Facility, David Geffen School of Medicine of the University of California at Los Angeles. The founders were identified by genotyping using direct PCR mouse tail lysis reagent (Viagen Biotechnology, Los Angeles, CA), proteinase K solution (Viagen Biotechnology), and the primers: 5′- CAGCCTTGGTCTCTGTCTACG-3′ and 5′-AACAGCACAAGGGGTTTTCTT-3′. These primers result in a 339 bp PCR product. In PCR, the primers selected for founder identification detects down to 1 copy of DNA constructs mixed with 100 ng of mouse genomic DNA, and thus are capable of unambiguously detecting the founders. The PCR primers were designed to test the fusion junction of opsin promoter and iNOS genes.

### Transgenic mouse production and analysis

The identification of founders, breeding of funders to generate F1 lines, initial screening of homozygotes and heterozygotes, and test breeding of possible homozygotes to WT to establish the homozygosity were all performed according to the published methods [15]–[17]. Especially, the final homozygosity confirmation was assured by test breeding to produce litters of at least 7 pups and all pups were tested positive for heterozygosity in that particular litter. Subsequent genotyping of heterozygous progeny resulting from expansion of colonies was performed using PCR analysis of tail genomic DNA.

### Genotyping

Approximately 0.5 cm long mouse tails were cut and placed in 200 µl tail lysis buffer containing 195 µl mouse tail direct lysis reagent (Viagen Biotechnology) and 4.8 µl proteinase k (Viagen Biotechnology) and incubated at 55°C for 26 hours. The lysed mixture was then heated to 85°C for 45 minutes to degrade the remaining proteinase k before PCR. TaqPCR Core Kit (Qiagen, Valencia, CA) was used to prepare the master mix, and 1–2 µl of supernatant from the tail lysis mixture was used for the PCR.

Primers for generating the 339 bp PCR fragment have been described in the first section of Materials and Methods.

### Western blot analysis

The isolated retinas were combined in a group of four and subjected to protein isolation (including low-output sonication) in Tris buffer (250 µl) containing a mixture of protease inhibitors (Roche Diagnostics, Indianapolis, IN). No detergent was added. Only a 20 µg portion of total extracted cytoplasmic protein (400 µg/4 retinas) was used per lane in Western blot. The protein was then fractionated by SDS-PAGE and the electrophoresed proteins were transferred by a semi-dry transfer unit (Hoefer, Inc. San Francisco, CA) to polyvinylidine fluoride (PVDF) membrane (Millipore, Billerica, MA). Nonspecific sites were blocked with 0.1%Tween 20 in 50 mM Tris-buffered saline (pH 7.4) throughout the staining procedure. For iNOS immunoblots (7.5% gel), the primary antibody used was rabbit polyclonal anti-iNOS (BD Biosciences, San Jose, CA; catalogue #, 610332; lot #, 18483; and dilution, 1∶2000) and secondary antibody was goat anti-rabbit IgG conjugated with biotin (Vector Laboratories, Burlingame, CA; catalogue #, BA-1000; lot #, W-1002; and dilution: 1∶200). For probing β-actin, primary antibody was mouse monoclonal IgG (Santa Cruz Biotechnology, Santa Cruz, CA; catalogue #, sc-47778; lot #, C3012; and dilution, 1∶1000) and the secondary antibody was goat anti-mouse IgG conjugated with biotin (Vector Laboratories; catalogue #, BA9200; lot #, W0206; and dilution: 1∶200). For nitrotyrosine immunoblots (15% gel), the primary antibody was rabbit polyclonal anti-nitrotyrosine (Millipore; catalogue #, 06-284; lot #, DAM 1748585; and dilution, 1∶200) and the secondary antibody was the same as that used for iNOS. For cytochrome c blots (15% gel), the primary antibody was monoclonal anti-mouse cytochrome c (BD Biosciences; catalogue #, 556433; lot #, 00736; dilution: 1∶300) and the secondary antibody was goat anti-mouse IgG conjugated with biotin (Vector Laboratories; same as the secondary antibody used for β-actin. Species specificity for all of the antibodies used includes mouse. After enhancement with a complex of peroxidase-conjugated biotin and avidin (ABC kit, Vector Laboratories), visualization was carried out using chromogenic 3,3′-diamino-benzidine/NiCl_2_ reagent (Sigma, St Louis, MO). Visualization was often carried out by Pierce enhanced chemiluminescence (ECL) Western blotting substrate (Thermo Scientific, Rockford, IL) to compare the results with chromogenic detection. The specificity of polyclonal anti-iNOS from BD Biosciences (catalogue #, 610332) was routinely checked for its specificity using a commercially available mouse macrophage + IFNγ/LPS lysate (BD Biosciences, catalogue # 611473; concentration, 1 mg/1 ml). The iNOS induced by these activated mouse macrophages has been cloned and characterized in the past [18], [19]. To confirm its specificity, the nitrotyrosine antibody was pre-blocked by incubating with authentic 3-nitrotyrosine (Sigma), the incubation should totally nullifies the reactivity of nitrotyrosine antibody.

### Immunohistochemical localization

Enucleated eyes devoid of corneas were fixed in 4% paraformaldehyde (PFA) at 4°C and then cryo preserved in 30% sucrose overnight (4°C) before embedding in optimal cutting temperature compound (OCT). The enucleated, intact eyes were also embedded in OCT first and the frozen sections were fixed with 4% PFA for 10 minutes at room temperature. Throughout the study, 10 µm sections were used for the staining. For localizing iNOS, non-antigenic sites were blocked with 2% goat serum and 1% BSA for 20 minutes. Slides were then incubated with the primary antibody, rabbit polyclonal anti-iNOS (BD Biosciences, the same primary used for the Western analysis; dilution, 1∶100) overnight at 4°C, and then with the secondary antibody, Alexa Fluor 568 goat anti-rabbit IgG (Molecular Probes/Invitrogen, Eugene, OR; catalogue #, A11036; lot #, 757102; and dilution, 1∶500) 1 hour at room temperature. Zeiss 510 laser scanning confocal microscope was used for the visualization. For localizing nitroyrosine, the primary antibody used was rabbit polyclonal anti-nitrotyrosine (Millipore; the same antibody as that for the Western probing; dilution, 1∶100) and secondary antibody was Alexa Fluor 488 goat anti-rabbit IgG (Molecular Probes/Invitrogen; catalogue #, A111034; lot #, 760000; and dilution, 1∶1000). All Alexa Fluor secondary antibodies were incubated 1 hour at room temperature. Zeiss 510 laser scanning confocal microscope was used for the visualization. For immunolocalization of nitrotyrosine, two control procedures were used to ascertain the specificity of the primary antibody: (a) primary antibody was replaced by PBS; (b) primary antibody was blocked by reacting with 5 mM commercial nitrotyrosine (Sigma) before staining.

### Detection of apoptosis

*In situ* end labeling of DNA fragments (TUNEL) was performed on 10 µm frozen sections. DNA strand breaks were detected by labeling the free 3′-OH ends of DNA fragments with the *In situ* Cell Death Detection kit, Tetramethyl rhodamine (TMR) red (Roche Diagnostics), following the manufacturer's specifications. Briefly, the sections were treated with permeabilization solution before incubating with terminal deoxynucleotidyl transferase which catalyzes reaction of labeled nucleotides to free 3′-OH DNA ends. Visualization was carried out using a Zeiss 510 laser scan confocal microscope with rhodamine 123 filter (excitation/emission, 480/550 nm).

### Morphologic evaluation

For light microscopy, eyes were enucleated without any injury and placed in Davidson's fixative (95% ethyl alcohol, 33.3 ml; 10% buffered formalin, 22.2 ml; glacial acetic acid,11.1 ml; and distilled water, 33.3 ml) for 24 hours before subjecting to the routine embedding. The steps of tissue processing are: 1) dehydration using graded strength of ethanol; 2) use of xylene as a clearing agent; 3) infiltration with paraffin and 4) orienting the tissue sample in paraffin and allowing it to solidify. To cut paraffin blocks, a thermostatically controlled water bath is used to float out tissue ribbons after sectioning. A drying oven (at 60 degree) was used to melt the paraffin before H&E staining.

Serial paraffin sections were cut through most of the eye, and selected sections were stained with hematoxylin and eosin (H&E). For each phenotype, multiple sections from at least two eyes collected from different animals were examined.

## Results

### Generation of transgenic mice with a murine opsin promoter fused to mouse iNOS cDNA in retinal photoreceptors

The opsin/iNOS transgene (Fig. 1) was constructed with opsin as the chosen promoter to drive the iNOS gene into the retinal photoreceptors. The parental CMV-driven iNOS expression plasmid was transfected into fetal retinal pigment epithelial cells to test protein expression before the single cell pronuclei injection. Four transgene-positive founders (#10, #24, #26, and #43) were subsequently identified from the live births. One of the female founder did not generate any offsprings. From the remaining three funders, two experimentally feasible heterozygous lines were established. Selected possible homozygotes were test bred to WT to subsequently identify and generate a colony of 16 confirmed homozygotes. These homozygotes were either used for the following experiments, or for generating homozygote colony. A pure heterozygote colony was also established for experimental purpose and also for preserving heterozygous lines.

### Characterization of iNOS transgenic mice

No developmental abnormality was noted in either homozygote or heterozygote offsprings. However, the breeding efficiency of homozygote to homozygote was noted to be less than normal. For all the experiments in the characterization of iNOS insertion and subsequent detection of retinal degeneration, 5 to 6 months old iNOS transgenic mice were used. The reason for selecting predominantly 5- to 6-month-old animals for experiments are twofold: 1) From the results reported by the existing literature in similar studies, P150 was reported to be the age when effects of the mutation become apparent in mouse opsin promoter-directed expression of simian virus 40 tumor antigen gene [20]; 2) The period was selected based on our own periodic screenings. We saw iNOS expression near P21, but the specificity of nitrotyrosine formation could not be ascertained until close to P150.

**Figure 2:**
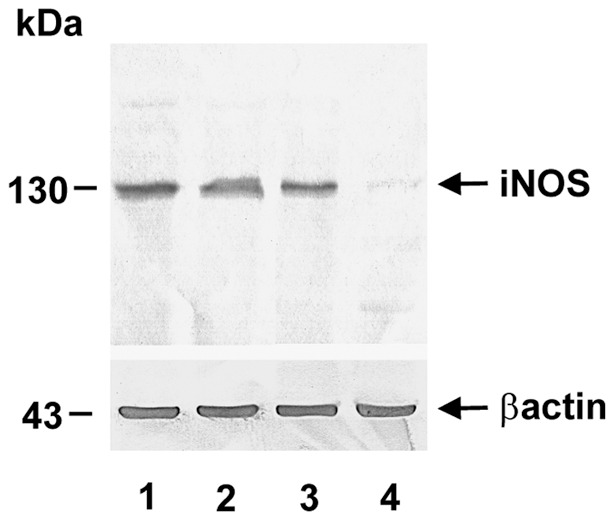
Confirmation of iNOS protein translation in the retina of transgenic mice. Following SDS-PAGE, the proteins were transferred to PVDF membrane for detecting with antibodies. Upper panel was probed with rabbit polyclonal anti-iNOS as primary antibody and goat anti-rabbit IgG conjugated with biotin as secondary antibody. For detecting β-actin in the lower panel, the primary antibody was mouse monoclonal anti-β-actin and goat anti-mouse Ig G conjugated with biotin. Lane 1: heterozygote, line a; lane 2: homozygote; lane 3: heterozygote, line b; and lane 4: C57BL/6 control mice.

To confirm the presence of iNOS in the retina, the Immunoblots were carried out on the total retinal homogenates probed with iNOS polyclonal antibody. A single, intense band was detected at 130 kDa (iNOS) using β-actin as loading control (Fig. 2). The molecular mass of the iNOS band was confirmed by the relative mobility (Rf) calculation using two protein markers: phosphorylase (148 kDa) and albumin (98 kDa). Both homozygotes and heterozygotes showed 130 kDa iNOS expression, with minimal difference in intensities.

**Figure 3:**
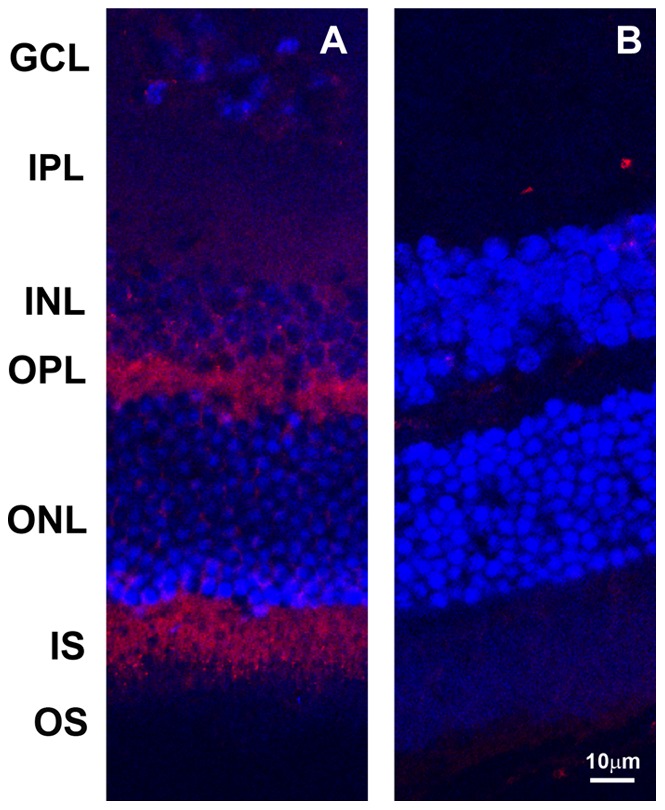
Immunohistochemical localization of inserted iNOS in the transgenic mouse retina. Rabbit polyclonal anti-iNOS (primary antibody) and Alexa Fluor 568 goat anti-rabbit IgG (secondary antibody) were used for the staining. A: retina from transgenic homozygote; and B: retina from C57BL/6 control. Retinal layers marked are: GCL: ganglion cell layer; INL: inner nuclear layer; OPL: outer plexiform layer; ONL: outer nuclear layer; and IS: photoreceptor inner segments. Note the intense staining of iNOS specifically in the IS and OPL and low intensity, dispersed staining in INL and IPL. In the control, only some low grade, non-specific staining is seen.

Translated iNOS protein level was further evaluated by localization of iNOS in the retina. In the confocal visualization, the opsin promoter-driven iNOS expression was intensely and specifically localized in photoreceptor inner segment (IS) and outer plexiform layer (OPL) specifically (Figs. 3A, 3B). The staining generally covers the entire layers of both IS and OPL. However, some low intensity, dispersed staining was also visible in inner nuclear layer (INL) and inner plexiform layer (IPL). The general feature of iNOS staining closely resembled those of a diphtheria toxin gene previously inserted by the same 4.4 kb rod opsin promoter [14]. Both homozygotes and heterozygotes presented the same staining patterns. The immunostaining of the control sections revealed only trace of red color in the outer segments from the secondary antibody, and no staining was seen in IS or OPL. For heterozygotes, the iNOS expression was also checked out by the immunoperoxidase staining with chromogenic visualization (data not shown) to compare with the results from confocal visualization. The specificity and intensity displayed by chromogenic staining appear to be similar to those from confocal visualization.

### Nitrosative stress in iNOS transgenic animals

**Figure 4:**
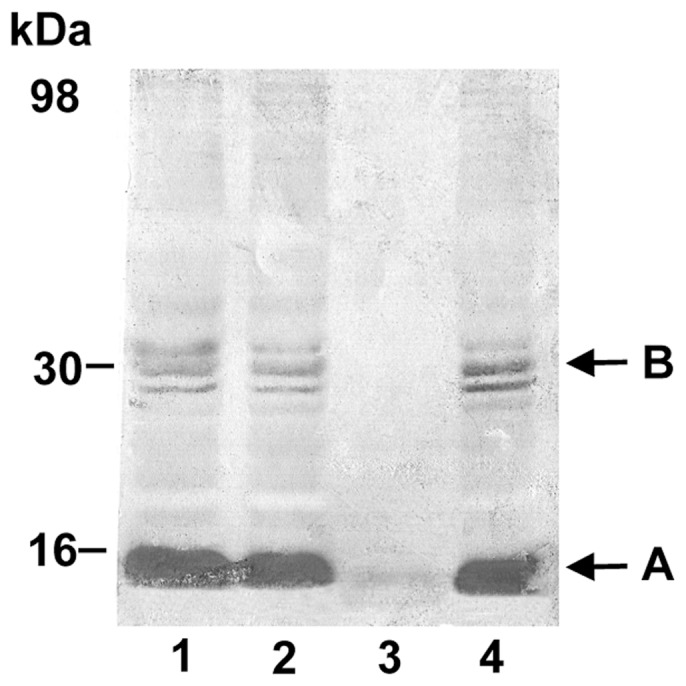
Detection of tyrosine-nitrated proteins in iNOS-overexpressed transgenic mouse retina. The total proteins were electrophoresed on 15% polyacrylamide gel and were probed with rabbit polyclonal anti-nitrotyrosine and biotinylated goat anti-rabbit IgG antibodies. Following enhancement with ABC kit, chromogenic visualization was used for the detection. Enhanced chemiluminescence (ECL)-based visualization was also carried out routinely for the comparison. Western blot analyses were carried out in triplicate and representative results are shown. Lane 1: heterozygote line a; lane 2: heterozygote line b; lane 3: C57BL/6 control; and lane 4: homozygote. Although there are several low-intensity tyrosine-nitrated bands in the background, the major nitrated protein pattern appears to be similar in all zygotes, with a major band near 16 kDa (A: nitrated cytochrome c monomer) and a doublet near 30 kDa (B: nitrated cytochrome c dimer). Further confirmation of these bands is presented in Figure 6.

Following establishment of the iNOS insertion, the pathogenic effect of iNOS was determined by evaluating NO/peroxynitrite-driven nitrosative stress in the iNOS transgenic retina. We sought specifically for the tyrosine-nitration in proteins, a stable oxidative/nitrosative post-translational nitration of tyrosine residues in the retina. In nitrotyrosine immunoblots of iNOS homozygotes and heterozygotes, a major nitrated band was seen near 15 kDa, indicative of nitrated cytochrome c [21]. The SDS-PAGE migration of the cytochrome c itself and its nitrated form are normally not resolved in one dimensional SDS-PAGE and therefore, appear as a single band. In Western analysis, nitrated cytochrome c from two different sources, EAU [21] and current study was exactly the same in their migration pattern characterized by the appearance of both cytochrome c monomer (15 kDa) and dimer (30 kDa) in the blot (data not shown). Cytochrome c is known for its polymorphism. Since, in the respiring cells, cytochrome c constantly undergoes oxidation/reduction cycles. Therefore, it is common to see oxidized form and reduced form co-exist in the cytochrome c analysis and this might be the source of cytochrome c doublet seen in Fig. 4 [22]. One less intense, but well-resolved doublet appeared lower than 36 kDa and close to 30 kDa (estimated by the relative mobility (Fig. 4). The molecular masses of this doublet match those of the nitrated cytochrome c dimer previously found in this laboratory from chemical nitration of commercial cytochrome c [21].

**Figure 5:**
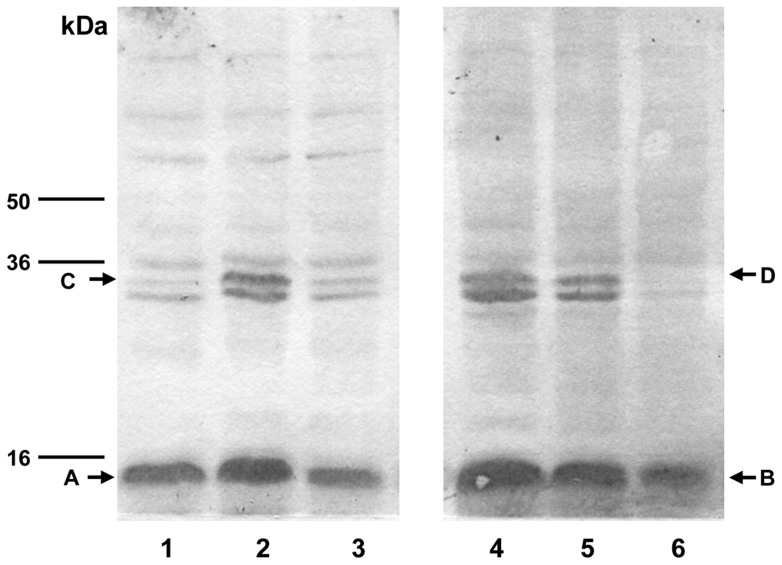
Confirmation of cytochrome c tyrosine-nitration and release in iNOS transgenic mice. The retina proteins were extracted with Tris buffer containing only protease inhibitors and no detergent. Total retina proteins were separated with15% polyacrylamide gel before transferring to PVDF membrane. Lanes 1, 2, and 3 were immunoblotted with monoclonal mouse cytochrome c antibody; lane 1: heterozygote line a; lane 2: homozygote; and lane 3: heterozygote line b. Lanes 4, 5, and 6 were immunoblotted with nitrotyrosine antibody; lane 4: homozygote; lane 5: heterozygote line a, and lane 6: heterozygote line b. A: cytochrome c; B: nitrated cytochrome c; C: cytochrome c dimer and D: nitrated cytochrome c dimer. Nitrated cytochrome c was presumably displaced from its binding site at the electron transport chain and was then released to the cytosol after a gentle mechanical disruption of the mitochondrial outer membrane by sonication.

The identification of the15 kDa band as nitrated cytochrome c was further shown by the two Western blots from the same membrane, but was probed separately with two different antibodies: lanes 1, 2, and 3, with monoclonal cytochrome c antibody and lanes 4, 5, and with polyclonal anti-nitrotyrosine detecting only a small part of the protein structure containing nitrated tyrosine (Fig. 5). These two blots demonstrated that the 15 kDa band is cytochrome c and this cytochrome c is also nitrated.

**Figure 6:**
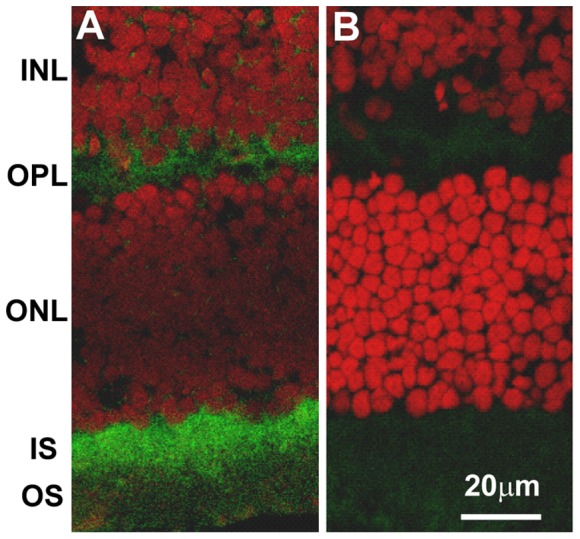
Immunohistochemical localization of tyrosine-nitrated proteins in transgenic mouse retina. Antibodies used for the confocal immunolocalization are rabbit polyclonal anti-nitrotyrosine and Alexa Fluor 488-conjugated goat anti-rabbit IgG (green). Propidium iodide (red) was used for the nuclear staining. The intense localization was seen specifically and uniformly in the photoreceptor inner segments (IS) and outer plexiform layer (OPL). These locations are known mitochondria-rich areas. A: retina section from homozygote; B: retina section from control. Retina layers labeled are: INL: inner nuclear layer; OPL: outer plexiform layer; ONL: outer nuclear layer; IS: photoreceptor inner segments and OS: photoreceptor outer segments.

The immunolocalization of nitrated proteins indicated that the prominent nitrated protein sites to be IS, and OPL (Fig. 6A). The sites of staining represent the mitochondria-populated area, especially in IS. In controls, low intensity green staining was seen in many areas of the retina and the staining appears diffused and non-specific (Fig. 6B). There is no detectable difference between homozygotes and heterozygotes in the intensity or the sites of positive staining.

### Apoptosis in the retina of iNOS transgenic mice

**Figure 7:**
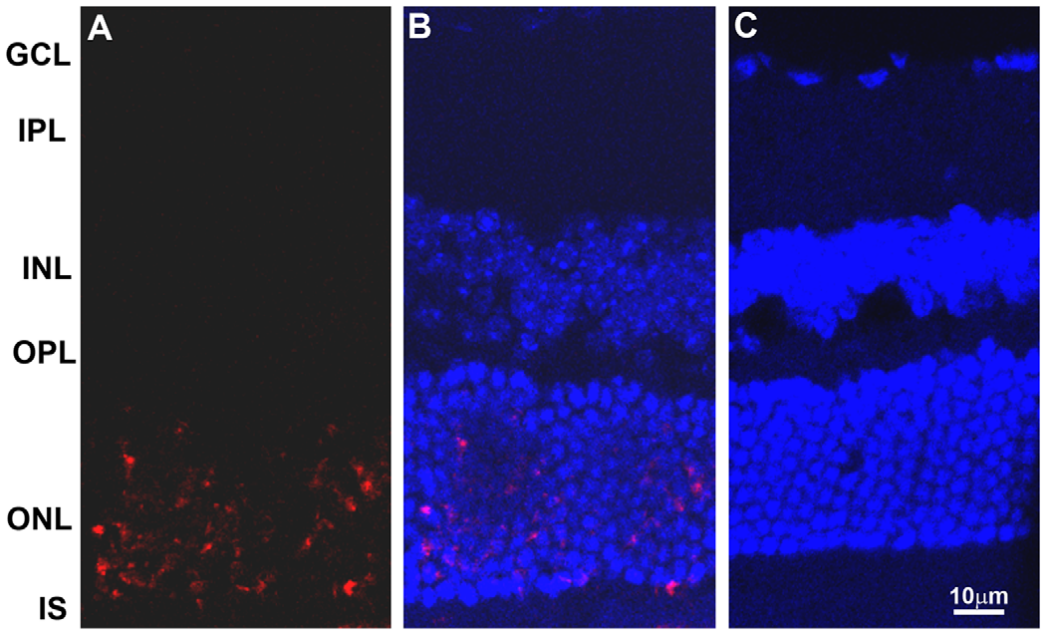
Detection of apoptotic cells in retina of iNOS transgenic mice. Retinal apoptosis was detected using *In Situ* Cell Death Detection Kit, TMR (trimethyl rhodamine) red, and the free 3′-OH from the DNA strand breaks are detected by modified nucleotides in an enzymatic reaction (TUNEL reaction). A: Apoptotic cells without nuclear staining; B: Apoptotic cells after DAPI staining; and C: C57BL/6 control retinal section with both TUNEL and DAPI staining. A large number of apoptotic cells were found specifically in the outer nuclear layer and none in the other retinal layers. A small number of apoptotic cells (3–4 cells) were routinely seen in the entire segment of control retinal sections. Substantially more apoptotic cells are visible in A without DAPI staining, since following merging with DAPI, some apoptotic cells were buried under the ONL nuclear staining.

In TUNEL staining, numerous apoptotic cells stained with red TMR were found exclusively in the nuclei of photoreceptor outer nuclear layer (ONL) and were absent in other layers (Fig. 7). The apoptotic cells were mostly individually separated, and no cluster was indicated. On merging with nuclear 4′,6-diamidino-2-phenylindole (DAPI) staining, some of the apoptotic cells were buried under the ONL nuclear staining. However, a substantial number of TMR cells were still visible. (Figs. 7A, 7B). The number of apoptotic cells in the confocal field (40× magnification) appears to be similar between homozygotes and heterozygotes. In controls, only 3-4 red-stained cells were visible in the entire segment of control retina.

After confirming the apoptosis in ONL, the morphologic changes in 6-month old homozygotes were evaluated. Using Davidson's fixative and undisturbed whole eyes, the H & E staining revealed no apparent major photoreceptor disruption. The retina was still generally preserved, except for some minor disorganized retinal layers, uneven thickness of ONL, and occasional retinal detachment (data not shown). We are continuing to evaluate the kinetics of photoreceptor dystrophy at different time periods on hetero- and homozygotes older than 6 months.

## Discussion

In this study, we used the mouse photoreceptor rod opsin promoter to direct overexpression of mouse iNOS in the photoreceptors. The efficacy of this promoter has previously been shown to be efficient and capable of inserting higher levels of transgene in the photoreceptors [14], [23], [24]. The translational efficiency from the inserted mouse iNOS was verified with a single130 kDa band in Western and confocal localization seen predominantly at photoreceptor IS and OPL. Initially, opsin promoter guides the reporter gene (or iNOS) to the photoreceptors, and there, the iNOS gene will follow the existing transcriptional factors/enhancers in the area to transcribe and translate, and the final localization is to follow the endogenous iNOS to nearby cytoplasmic locations, such as IS and OPL, where iNOS is normally induced and reside [25]. In iNOS staining, besides the major IS and OPL localization, there are also some scattered, low intensity staining in INL and IPL (Fig. 3). Previously, using this rod opsin promoter fused to lac *Z* transgene, intense X-Gal staining was found in the layers IS and OPL, but low intensity diffused staining was also noted in INL and IPL [14] with close similarity to this study. Therefore, the current rod opsin promoter will effectively insert different reporter genes in the photoreceptors, but the transgene delivery is not always completely specific.

The iNOS-derived pathologic effects were evaluated by detecting the oxidative/nitrosative products, focusing on the post-translationally modified tyrosine- nitrated proteins. In confocal localization, the major sites of protein nitration were in photoreceptors IS and OPL where the mitochondria are densely populated [26]. High output of NO generated from iNOS, as in the present case, rapidly scavenges superoxide and generates peroxynitrite, a source of most prominent nitrosative stress in the tissue. Tyrosine-nitration in proteins reflects specifically this reactive pathway of peroxynitrite [27], [28]. In addition, peoxynitrite, the most potent biological oxidant known to date, once formed further amplifies the oxidative and nitrosative stresses [27], [28].

For evaluating the formation of protein tyrosine-nitration, protein extraction was performed using lysis buffer (containing Tris and protease inhibitors only), suitable for extracting only the soluble cytoplasmic proteins, exclusive of cytoskeletal- or membrane-bound proteins. No detergent was added in this lysis buffer. From extracted cytosolic proteins, intense nitrated protein bands were detected at 15 kDa and 30 kDa, consistent with cytochrome c monomer and dimer respectively. The identity of these bands was further confirmed by blotting the same cytosolic proteins with 1) cytochrome c antibody detecting both cytochrome c monomer and dimer, and 2) nitrotyrosine antibody detecting the nitrated tyrosine structure in any protein. The results indicated that the 15 and 30 kDa bands were indeed cytochrome c and that it was also tyrosine-nitrated (Figs. 4, 5A, and 5B).

Cytochrome c is a multi-functional enzyme involving in both life and death of the cell. It participates in electron transfer reaction as part of the mitochondrial electron transport chain and is thus an important molecule for energy production process [29]. However, it also participates in apoptosome formation leading to the eventual cellular apoptosis [29]. These seemingly contradictory functions of cytochrome c, therefore, are tightly regulated by several cell signaling pathways. However, when the oxidative stress overwhelms the sustaining of homeostasis, programmed cell death occurs through intrinsic (mitochondrial) apoptosis initiated by the release of cytochrome c [29].

A functional cytochrome c normally binds electrostatically to both mitochondrial respiratory complex III and IV in the mitochondrial inner membranes. It is therefore, stable to gentle mechanical disruption of the retina, but sensitive to detergents that are added into the lysis buffer and capable of digesting cellular membranes [30]. No detergent was added in the protein extraction in this study. Further, unlike other respiratory chain complexes, the cytochrome c molecule embedded in the inner membrane faces intermembrane space rather than matrix, and substantial portion of the structure is protruding outside the inner membrane boundary into the intermembrane space. This rather unusual spatial arrangement makes cytochrome c considerably more vulnerable to peroxynitrite attacks than other respiratory chain complexes residing in the inner membranes [30]. Previous reports have also revealed that when cytochrome c was released from permeabilized mitochondria, it was often found that cytochrome c was already dissociated from its native location within the hierarchically arranged mitochondrial respiratory complexes before the pathologic membrane rupture [30]–[32]. Therefore, it appears that once the cytochrome c is nitrated, it is likely that cytochrome c is already displaced from its original binding site in the inner membrane; possibly due to the structural alteration with added NO_2_ group. Thus, during the protein extraction, a gentle mechanical rupture of mitochondrial outer membranes releases cytochrome c to the supernatant to be detected by immunoblot [21]. Therefore, we found a substantial release of cytochrome c in iNOS overexpressed retina under basal conditions without any stimulation or insult.

It is now generally accepted that in the mitochondrial death pathway, a key step in the apoptotic cascade involves the release of cytochrome c into the cytosol where it binds with apoptotic protease-activating factor 1 (Apaf-1). Binding of cytochrome c results in an increased affinity of the complex for dATP leading to the formation of apoptosome [33]. The pro-caspase-9 is then recruited and cleaved by apoptosome to its active form, caspase -9. Caspase-9 then initiates the apoptosis by acting as the cleavage factor for caspase-3, its cleaved subunits p20 and p17 serve as executioners of apoptosis [34], [35].

The retinal photoreceptor apoptosis occurs in various retinal diseases [36]–[38]. Despite the difference in their causes, it appears that in several eye diseases, oxidative stress-mediated apoptosis is the cause of the initial mechanism of retinal degeneration [36]–[38]. The route by which the initial indicate of apoptosis subsequently leads to loss of the entire photoreceptor is as yet to be fully elucidated. Using TUNEL, the retinal apoptosis was detected in iNOS transgenic mice (Fig. 7A, 7B, and 7C). A large number of apoptotic cells were found in photoreceptors only. This implies that apoptosis was a direct consequence of iNOS-overexpression for two reasons: 1) the appearance of apoptotic lesions follows the localization pattern of iNOS protein specifically in photoreceptors; and 2) no apoptotic cells were detected when the same opsin promoter-expression construct was used to express *LacZ* [14], or rod and cone transducin [39]. Our apoptosis results from iNOS overexpression, thus indicate that the iNOS derived from the undue causes and its susbsequent photoreceptor apoptosis may represent initiation of photoreceptor degeneration through apoptosis and these apoptosis pathways often accompany cytochrome c release [39]–[41].

Although low constitutive levels of previously known nNOS and eNOS are expressed in iNOS transgenic mice, there is evidence to suggest that these isoforms do not confer a substantial effect in the retinal degeneration. The reasons are summarized as follows: 1) Constitutive nNOS/eNOS and iNOS have distinctively different functions. Neuronal NOS has been implicated in synaptic plasticity, central control of blood pressure, and neurotransmission; whereas, eNOS-derived NO is a physiological vasodilator [42]–[45]. On the contrary, iNOS is up-regulated in various types of inflammatory disease, and the large output of NO mediates various aspects of inflammation [10]; 2) In age-matched controls which regularly express constitutive levels of eNOS/nNOS, the positive results have never been found in detection of retinal degenerative parameters, such as nitrotyrosine and apoptosis.

In spite of these prominent degenerative effects of iNOS displayed, there is not yet a gross alteration of retinal morphology observed in the photoreceptors of iNOS transgenic mice. In a future study, visual functional tests, such as ERG, should be carried out on these transgenic animals. One likely reason for not seeing the photoreceptor loss could be the upregulation of protective mechanisms, such as crystallins [34] is in place as soon as the iNOS stress signal is detected. Therefore, double mutants with photoreceptor-specific iNOS overexpression and concomitant αA crystallin deficiency [34] (iNOS^+/+^/αAcrystallin ^−/−^) might increase the vulnerability of iNOS-overexpressed photoreceptors to degeneration. The generation of this double mutant by crossing iNOS^+/+^ with αA crystallin^−/−^ is currently underway in this laboratory. A similar approach has recently been taken to exacerbate the toxicity of iNOS in the heart [46]. Transgenic mice with cardio specific overexpression of iNOS did not develop heart failure. However, double mutants with cardiomyocyte-specific overexpression and concomitant myoglobin deficiency (iNOS^+/+^/myo^−/−^) developed definitive sign of heart failure [46].

In summary, we have generated a pathologic phenotype with sustained iNOS expression in the photoreceptors. These transgenic mice allow us to evaluate the genuine iNOS effect totally discerned from the immune mediators by which iNOS is normally induced and associated in inflammation. At this stage of investigation, our results revealed that cytochrome c was tyrosine-nitrated and released from its normal binding site in the mitochondrial inner membrane. In the past, the occurrence of protein nitration through the iNOS oxidative/nitrosative pathway has been well documented [6], [41], [47]. In this study, the localization of protein nitration was found specifically in inner segments particularly rich in mitochondria. Therefore, we have an initial clear indication of cellular damage derived from sustained iNOS expression. Undoubtedly, the cellular events that result in mitochondriopathy and/or cell death, such as apoptosis [29], [40] are more complex, and further investigation is warranted for these studies.
